# Characterization of graphene-filled fluoropolymer coatings for condensing heat exchangers

**DOI:** 10.1177/00219983211037053

**Published:** 2021-08-10

**Authors:** Mitchell Cierpisz, Joselyne McPhedran, Youliang He, Afsaneh Edrisy

**Affiliations:** 1 CanmetMATERIALS, Natural Resources Canada, 302492 CanmetMATERIALS, Canada; 2 Department of Mechanical, Automotive and Materials Engineering, University of Windsor, Canada

**Keywords:** Condensing heat exchanger, fluoropolymer composite coating, graphene, thermal conductivity, corrosion resistance

## Abstract

Condensing heat exchangers are thermal devices subjected to extremely corrosive environments due to the formation of acidic condensates on the heat-exchange elements during service. To protect the heat exchangers from chemical attack, perfluoroalkoxy (PFA) coating has been applied as a barrier layer onto the surfaces of the heat-exchange elements to prevent corrosion. However, PFA has intrinsically poor thermal conductivity, and low wear resistance; thus, it is not naturally a good material for heat exchanger application. In this study, graphene nanoplatelets (GNPs) are incorporated into PFA powder as coating materials to improve the thermal properties of the fluoropolymer, for condensing heat exchangers application. Two grades of GNPs (8 nm and 60 nm layer thickness) are tested to evaluate the effect of graphene addition on the thermal, adhesion, electrical, and wear properties of the composites, which are compared to those mixed with multi-walled carbon nanotubes (MWCNTs). The results showed that both grades of GNPs significantly increased the thermal conductivity, i.e., ∼8× that of the virgin PFA. The composites incorporated with both grades of GNPs also demonstrated good coating adhesion strength and wear resistance, as well as excellent corrosion resistance. The composite filled with MWCNTs exhibited poor surface finish and minimal improvement in thermal performance.

## Introduction

Waste heat recovery is a key technology for the processing industry to reduce energy waste by recycling the waste heat contained in hot exhaust streams, consisting of up to 70% of the total energy from the input fuel.^1^ Low-temperature waste heat, i.e. temperatures below ∼230°C, accounts for ∼60% of all unrecovered waste heat,^1^ since low-grade waste heat is not economically viable to recover using conventional heat recovery techniques. This is due to the low work potential of low-grade waste heat and the high cost associated with the rapid corrosion of the heat recovery devices. To maximize the recoverable energy, it is necessary to recover both the latent and sensible heats, which requires the exhaust streams to be cooled below their dew points. At these temperatures, corrosive acids condense on the heat-exchange element surfaces, causing severe corrosion and premature failure of the heat recovery equipment.^1,2^ Traditional heat exchangers made of low-cost metals (e.g. aluminum, stainless steel, copper, etc.) are not suitable for the condensing environment due to the catastrophic chemical attack on the heat-exchange elements. Exotic metals such as titanium, tantalum, niobium, etc., may withstand the corrosive conditions, but they are not economically viable for low-grade waste heat recovery.

Recent studies have illustrated the suitability of fluoropolymer-based coatings as a barrier layer to protect heat-exchange elements from chemical attack.^2,3^ Perfluoroalkoxy (PFA) is an attractive coating material for condensing heat exchangers due to its superior corrosion resistance, relatively high operating temperatures (up to 260°C), and its melt-processable characteristics.^2–4^ However, PFA is not naturally suitable for heat exchanger applications because of its low thermal conductivity, and susceptibility to wear.^2^ High thermal conductivity is required to efficiently conduct the heat, while wear resistance is desired in applications where the heat-exchange elements are subject to the wear caused by solid particles from the exhaust streams. A variety of filler materials, e.g. graphite, silicon carbide, boron nitride, aluminum oxide, etc., have been employed to improve the thermal, mechanical, and tribological properties of the fluoropolymer.^3^ It has been shown that a PFA-based composite with 20 wt% boron nitride (thermal conductivity = 30–33 Wm^−1^K^−1^) showed the most significant improvement in thermal property, i.e. increasing the thermal conductivity to ∼4× that of the pure PFA (thermal conductivity: 0.19–0.21 Wm^−1^K^−1^).^3^ However, this is still far from the thermal conductivity of common metals. Further improvement in the thermal properties of the PFA composite is still needed to achieve higher heat-exchange efficiency.

The filler materials suitable for condensing heat exchangers must have similar corrosion resistance to the fluoropolymer, and they must also have high thermal conductivity. Graphene, a two-dimensional sheet of carbon atoms, sp^2^-bonded into a hexagonal arrangement,^5^ is known for its extraordinary thermal, mechanical and electrical properties.^6^ The highest recorded thermal conductivity of single-layer graphene (at room temperature) is 5300 Wm^−1^K^−1^.^7^ The exceptional electrical and thermal properties of graphene are attributed to its two-dimensional crystal structure in which the stiff covalent bonding among the carbon atoms generates a superior electron carrier space,^6^ and makes thermal vibration propagate with minimal dissipation under non-equilibrium conditions.^8^ Monolayer graphene displays higher thermal properties compared to multi-layer graphene^9^; when the number of graphene layers exceeds 10, the thermal conductivity approaches that of bulk graphite.^10^ Nevertheless, the overall performance of graphene-filled polymer composites is dependent on the quality, thickness, orientation of the graphene particles in the matrix^11^ as well as on the amount of filler and the microstructure of the composite resulted from the fabrication process. Multi-walled carbon nanotubes (MWCNTs), another form of carbon, are also commonly used in thermal management applications.^12^ The thermal conductivity of MWCNTs at room temperature is 3000 Wm^−1^K^−1^.^7^ However, the properties of graphene and MWCNTs are dependent on the direction.^13^ Due to their superior thermal properties, both graphene and MWCNTs are considered as candidate filler materials for this study.

This paper investigates the effect of graphene and MWCNTs, as potential fillers, on the thermal, adhesion and tribological performance of PFA-based composite coatings. The introduction of filler materials with high thermal conductivities to a polymer matrix is intended to enhance the thermal properties of the composite.^14–16^ Two grades of graphene nanoplatelets (8 nm and 60 nm layer thickness) were incorporated into PFA to examine the effect of graphene layer thickness on the properties of the composites. Microscratch tests were conducted on the composite coatings to evaluate the adhesion strength of the coatings to the substrates and the coatings abrasion resistance. Wear tests (both ambient and elevated temperatures) were performed to assess the coatings’ durability and robustness. Immersion corrosion tests in heated sulfuric acid solution were conducted to evaluate the corrosion resistance of the composite coatings in very corrosive environments similar to those encountered by condensing heat exchangers. Optical/laser microscopy and scanning electron microscopy (SEM) were performed to analyze the filler distribution within the coating and characterize the worn surfaces. Suitable filler materials were then selected based on their overall performance, i.e. thermal conductivity, adhesion strength, wear resistance, corrosion resistance, cost, etc. The results of this research provided practical guidance on the selection of suitable coating materials for condensing heat exchangers.

## Experimental procedure

### Materials

Neoflon AC-5600 PFA powder (Daikin Inc., Japan) was used as the polymer matrix. The powder has a nominal particle size of ∼44 μm (325 mesh) and a true density of 2.15 g/cm^3^. GNP and MWCNT powders (Graphene Supermarket, USA) were selected as the filler materials, with true densities of 2.25 g/cm^3^ and 2.1 g/cm^3^, respectively.^17,18^ Two grades of GNP and one grade of MWCNT powders were incorporated into the PFA powder to form the composites. Table 1 lists some properties of the three filler powders. The composite powders were prepared by mixing 1 to 20% (weight percentage) of the filler powders with the PFA powder using a tumbler mixer at room temperature for 1 hour.

**Table 1.** Properties of filler materials.^7,19,20^

Material property	AO-2 GNPs	AO-4 GNPs	MWCNTs
Young’s modulus (GPa)	∼1100	∼1100	800–950
Fracture strength (GPa)	125	125	63–150
Thermal conductivity (Wm^−1^K^−1^)	25–470^a^	25–470	∼3000
Specific surface area (m^2^/g)	>15	≤40	–
Diameter (nm)	–	–	50–85
Length (µm)	–	–	10–15
Average flake (lateral) size (µm)	∼5	∼7	–
Average flake thickness (nm)	8 (20–30 monolayers)	60	–
Aspect ratio	∼600	∼100	100–300

GNPs: graphene nanoplatelets; MWCNTs: multi-walled carbon nanotubes.

^a^Due to the number of graphene layers, the thermal conductivity listed is for bulk graphite rather than monolayer graphene.

### Thermal property measurements

**Figure 1.**
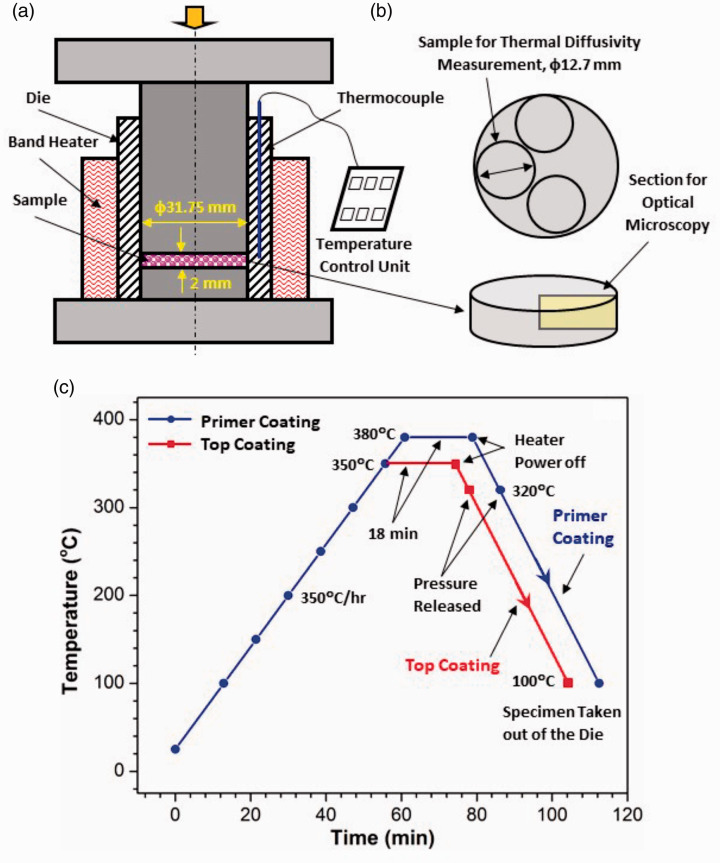
Schematics of the compression molding setup and the curing schemes used for the primer and top coatings: (a) compression molding setup, (b) sample cutting, (c) curing schemes.

The mixed composite powders were compression molded under a pressure of 10 MPa at 350°C (Figure 1(a)). Disk samples (φ12.7 mm × 2 mm) were sectioned from the molded disks (φ 31.75 mm × 2 mm) for thermal conductivity measurements (Figure 1(b)). The specific heat was determined using a differential scanning calorimeter with sapphire as the reference material (ASTM E-1269-11).^21^ The thermal diffusivity was measured using a laser flash method (Netzsch LFA 457 Microflash, Germany) according to ASTM E1461-01.^22^ The specific heat and thermal diffusivity were measured over a temperature range from 25 to 250°C. The thermal conductivity (λ) was then calculated using the relationship among the thermal conductivity, specific heat (C_p_), thermal diffusivity (α), and density (ρ)^23^:

(1) $${\text{λ=ρC}}_{\text{p}}\text{α}$$

Optical microscopy was performed on compression-molded samples to determine the size and distribution of the filler materials within the polymer matrix. Analysis of the particle distribution was conducted using the ImageJ software.^24^

### Electrical resistivity

Surface electrical resistivity of the composite disks was measured using a four-probe resistivity measuring system (Lucas Signatone, USA). Five measurements were taken on each sample and the average value was reported as the surface resistivity. For samples with very low electrical conductivity, the resistivity may not be measurable as it was out of the measuring limits of the system.

### Coating Application

The mixed composite powders were applied onto 316 stainless steel coupons (50 mm × 25 mm × 3 mm) through electrostatic powder spray. A small hole (Φ5 mm) was drilled in each coupon to facilitate the coating process and the subsequent immersion corrosion test. Sharp edges and corners were removed (rounded) to ensure smooth coating coverage in these regions. Before coating, the coupons were sandblasted using glass beads to prepare the surfaces for coating. A Daikin Neoflon ACP-5909BK primer powder ( ~50 μm particle size) was first applied onto the coupons and cured. The composite powders were then applied on top of the primed coupons and cured again. The curing schemes for both the primer and top coating are shown in Figure 1(c). The coating thickness (including the primer) was 150–250 μm, and usually 3–5 coats were required to reach these thicknesses. Microstructure analysis was performed on the cross sections of the coated coupons to determine the area fraction and distribution of the filler material within the composite coating.

### Corrosion test

The coated samples were immersed in an 80% sulfuric acid solution heated at 85°C for a total of 1500 hours. The mass of each sample was recorded before and after immersion to observe any mass gain or loss. Visual observation of the coatings was also performed to observe any colour change after immersion.

### Microscratch and wear tests

To evaluate the adhesion strength of the coating, microscratch test was performed under progressive loading (0 - 28 N) at a lateral speed of 1 mm/min over a 5-mm scratch track using a diamond Rockwell stylus (200 µm radius). The generated scratches were examined under SEM (ESEM, FEI-Quanta 200FEG) to assess the mechanism of failure. Ball-on-disk wear test was also conducted to assess the wear resistance of the coating. The (unlubricated) wear test was performed at both ambient (25°C) and elevated (200°C) temperatures using 10-mm diameter steel balls. An applied load of 5 N at a sliding speed of 0.1 m/s was utilized during the test, and each test ran for 8 hours. The wear track profiles were analyzed using optical/laser microscopy (Keyence, VK-X100) to determine the removed area. The volumetric wear rate ( $\tilde{W}$ ) was then calculated using the worn area (A), the wear track radius (R) and the sliding distance (S)^25^:

(2) $$\tilde{W}=2\pi \mathrm{RA}/S$$

The tangential (F_t_) and normal forces (F_n_), which were recorded during the test, were utilised to determine the coefficient of friction (µ = F_t_/F_n_). The variation of the coefficient of friction with respect to the sliding distance was plotted for each test condition, and the average coefficient of friction for each sample was calculated from those in the steady state.

## Results and discussion

### Distributions of the fillers in the compression-molded samples

**Figure 2.**
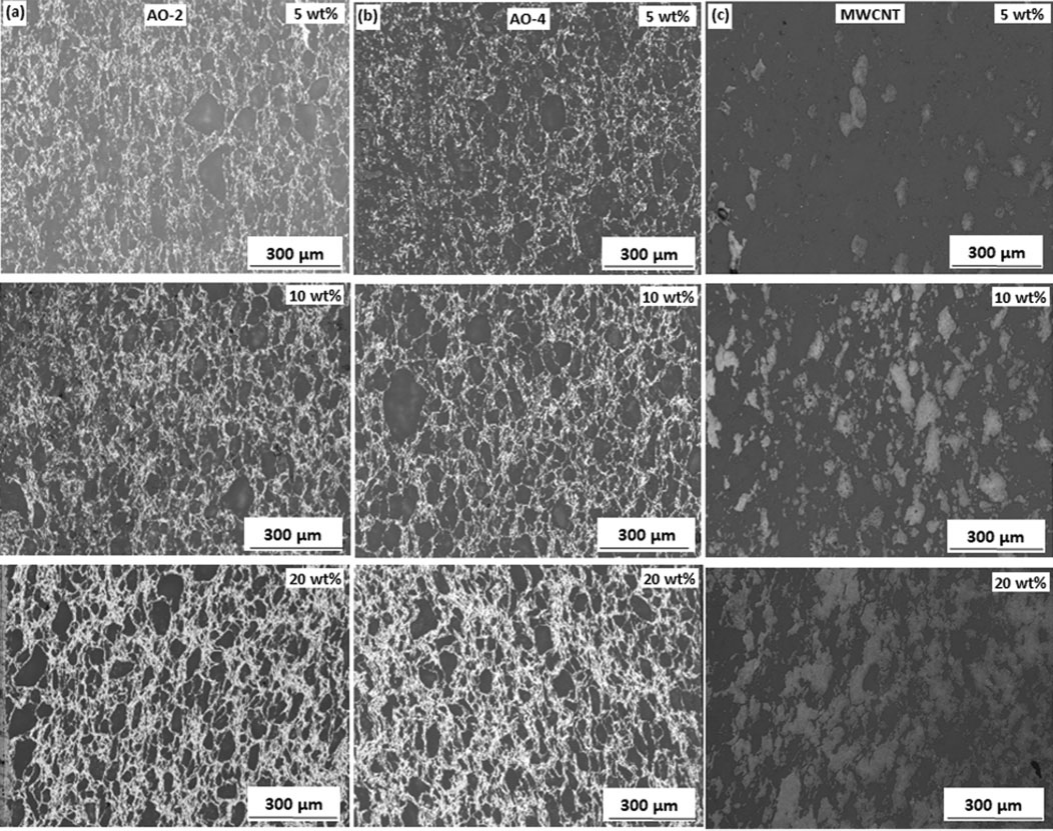
Distributions of the fillers within the PFA matrix (5, 10, and 20 wt%) and the variation of the filler area fractions with respect to the filler loading in the compression-molded samples: (a) AO-2 GNP filled, (b) AO-4 GNP filled, (c) MWCNT filled, (d) variation of the filler area fractions with respect to the filler amount. The filler is in light gray and the PFA is in dark gray. Compression was applied in the horizontal direction in these figures.

**Figure 3.**
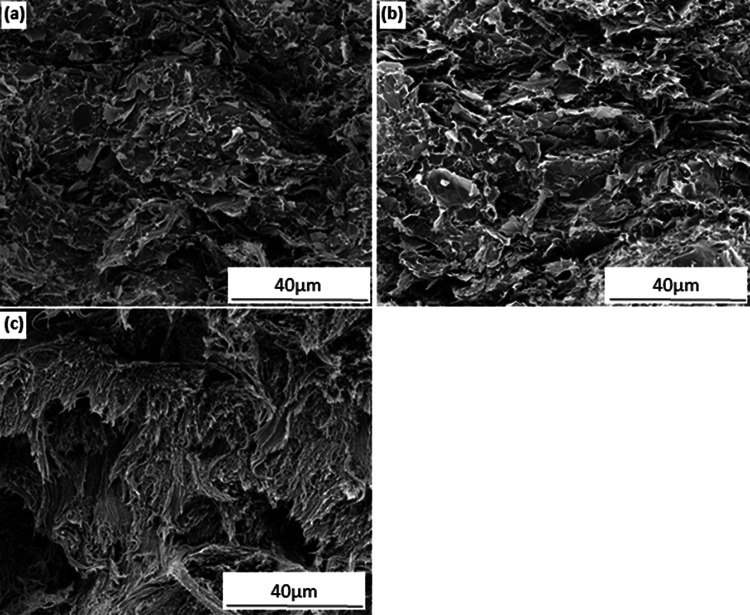
SEM images showing the fracture surfaces of the compression-molded samples with 20 wt% of different fillers within the PFA matrix: (a) AO-2 GNP filled, (b) AO-4 GNP filled, (c) MWCNT filled.

Figure 2 illustrates the distributions of the fillers in the compression-molded samples (5, 10 and 20 wt%) and the variations of the area fractions of the various fillers with respect to the filler loading. Generally, the filler area fraction increases with the filler loading (Figure 2(d)), which is expected to lead to an increase of both the thermal and electrical conductivity. When the filler content reached 20 wt%, both grades of GNP fillers form well-established networks within the PFA matrix, whereas the MWCNT-filled sample does not form a network between filler particles (the filler aggregated in the matrix). However, the area fraction of the filler particles for all the three samples are quite close (i.e. 33% - 38%). This can be seen more clearly in the SEM images (Figure 3) under higher magnifications. These images are the fracture surfaces of the compression-molded samples which show randomly distributed but connected graphene sheets embedded in the matrix (Figure 3(a) and (b)) or bundled MWCNT fibers (Figure 3(c)).

It is well known that in addition to the characteristics of the filler and matrix, the degree of dispersion and the interaction between the filler and matrix at their interface also have a great effect on both the mechanical and thermal properties. The mixing method directly influences the dispersion of the particles within the matrix and the interface strength between the filler and the matrix. The discrepancy in network formation in GNP and MWCNT samples shown above is likely due to the tumble mixing method used in this study. Apparently, uniform dispersion of the MWCNT is difficult through tumbling. Greater improvements in thermal performance of graphene polymer composites have been previously reported in literature through sonication^26^ and ball milling^27^ mixing methods. Improvement to the interface between the polymer and filler materials through ball milling, functionalization, as well as multiple other methods, have been shown to lead to enhancement in thermal and mechanical properties as well.^28–34^

### Thermal conductivity

**Figure 4.**
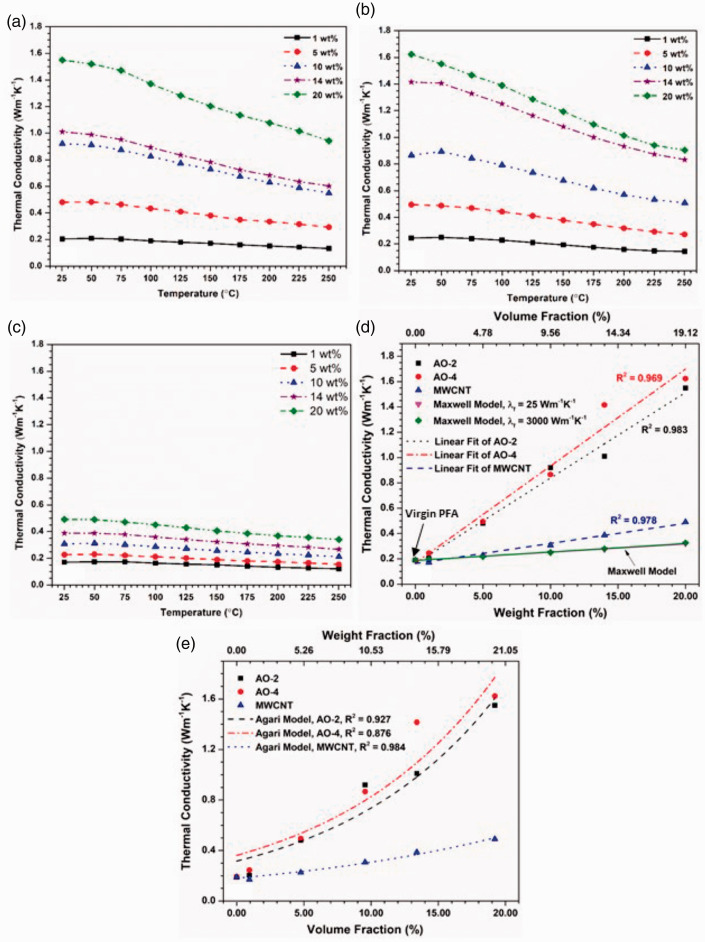
Thermal conductivities of the composite materials: (a) filled with AO-2 GNPs, (b) filled with AO-4 GNPs, (c) filled with MWCNTs, (d) the relationship between the thermal conductivity and the filler amount at room temperature as compared to the Maxwell model, (e) the thermal conductivity at room temperature fitted using the Agari model.

The thermal conductivities of the composites, calculated from the measured thermal diffusivity and specific heat (Equation (1)), are plotted against the temperature and filler amount in Figure 4. For all the composites, the thermal conductivity increases with the filler amount and decreases with the temperature. However, the increase of the thermal conductivity by incorporating GNPs is much more significant than by filling MWCNTs. At room temperature, the maximum thermal conductivity reaches ∼1.6 WK^−1^m^−1^, which is ∼8× of the pure PFA and ∼2× of graphite or boron nitride filled composites^2,3^ when 20 wt% of graphene (both grades) is added, while adding the same amount of MWCNTs, the thermal conductivity is only increased to ∼0.5 WK^−1^m^−1^. Thus, incorporating GNPs into PFA is much more effective than adding MWCNTs in enhancing thermal conductivity. Of the two grades of GNPs, the AO-4 filled composites show consistently higher thermal conductivities than those filled with AO-2 GNPs under the same weight percentages. Therefore, larger GNP filler particles (AO-4 grade) resulted in greater improvements in the composites thermal performance compared to those filled with smaller GNP particles (AO-2 grade). Previous studies^35,36^ have shown that the graphene layer thickness and the lateral size of the GNPs have a profound effect on the thermal conductivity: the larger the graphene layer thickness and lateral size, the higher the thermal conductivity. Larger GNP filler materials require fewer particles to achieve the same filler weight percent compared to smaller particles, resulting in fewer GNP-matrix interfaces. These interfaces are prone to interfacial thermal resistances and lead to lower thermal conductivities within the composite.^35^ As a result, the incorporation of GNPs with a larger graphene layer thickness (AO-4) can more effectively enhance the thermal conductivity.

Graphene is a 2D material and its in-plane thermal conductivity is among the highest of any known material.^13^ However, when incorporated into polymer with random orientations, the thermal conductivity of the composite can only be moderately improved. There is a thermal percolation threshold^37^ before which the heat transport is governed by the thermal conductivities of the polymer matrix and the filler as well as the thermal contact resistance between the filler and the matrix. Even when the thermal percolation is reached, i.e. an inter-connected filler network is established, there is still large inter-filler thermal contact resistance, which limits the propagation of the heat flux through the percolated filler network. Thus, the thermal conductivity is not expected to have comparable values as the filler, which is different from the percolation of electrical conductivity.^37^

Figure 4(d) shows the relationships between the filler fraction and the thermal conductivity of all three filler materials at room temperature. It is seen that for all the three fillers, the thermal conductivity approximately follows linear relations with respect to the loading fraction. However, these relations cannot be described by the Maxwell model^38^ even when very different values of the filler thermal conductivity (from 25 to 3000 Wm^−1^k^−1^) are used. This is because the Maxwell model assumes that the filler has a sphere shape which does not apply to the present study (the GNP and MWCNT fillers have large aspect ratios). It is also noted that at 14 wt% filler content, the thermal conductivity of the AO-4 filled composite is considerably higher than that filled with AO-2 (this is also noted in Figure 4(a) and (b)). This large discrepancy may be caused by the uneven dispersion of the filler in the composite. The section of the AO-4 sample might contain a locally higher concentration of filler than the average, while the AO-2 section might have a locally lower content than the average.

A number of non-linear models have also been proposed to predict the thermal conductivity of composite from the volume fraction of the filler material, e.g. Agari and Uno^39^, Mamunya et al.^15^, and Zhang et al.^40^ Some of these models need to know the thermal conductivity at the filling limit or at the percolation threshold to evaluate the composite thermal conductivity. Apparently, the maximum amount of filler (∼19% volume fraction) added in this study did not reach the percolation threshold yet, since the onset of percolation usually shows a super-linear relation.^37^ Thus the Agari model, which does not need the percolation threshold, was used to fit the experimental data at room temperature. From Figure 4(e) it is seen that the Agari model fits the data of the MWCNT filler very well (adjusted R^2^ = 0.984), but that of the AO-4 filler can only be reasonably well fitted (adjusted R^2^ = 0.876).

It should also be noted that the processing method (compression molding) used to prepare the samples resulted in an almost random distribution of the filler particles. Thus, the filler materials were not aligned in their preferred direction to optimize the thermal conductivity in the direction of the thermal flow. As a result, the thermal conductivity values are significantly lower than the theoretical values of the carbon materials.^41–43^

### Electrical resistivity

**Figure 5.**
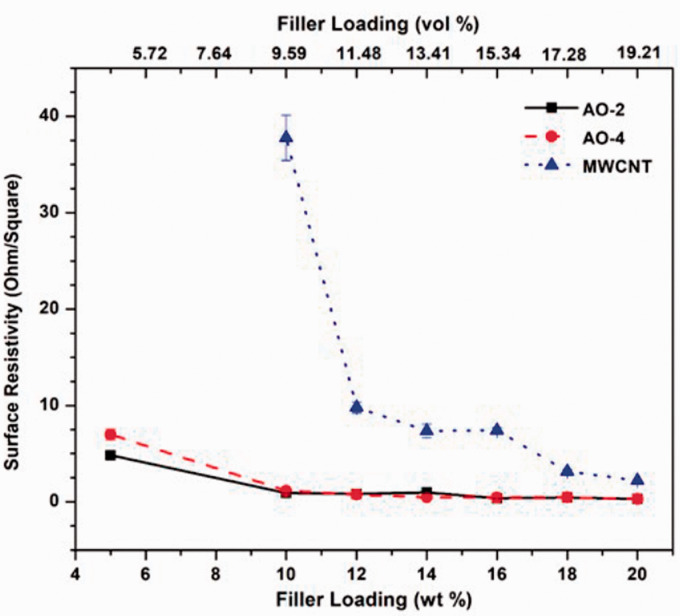
Relationship between the surface electrical resistivity and the filler loading.

Electrical resistivity measurements were performed on the surfaces of the compression-molded disks, and the results are shown in Figure 5. The samples loaded with both grades of GNPs yielded much lower surface resistivities than those loaded with MWCNTs, especially when the filler amount is smaller than 18 wt%. When the MWCNT filler is less than 10 wt%, the resistivity is very high and exceeds the measuring limits, thus the resistivity value could not be obtained. Similarly, the resistivity of the pure PFA and those of the samples containing 1 wt% GNPs or MWCNTs were also out of the measuring limits of the system, thus could not be obtained. As mentioned previously, due to the formation of randomly distributed filler networks in the graphene-filled composites, the surface electrical conductivity of the composite can be largely enhanced since these networks form the pathways for the transfer of the electrons^44^. The MWCNT-filled samples, on the other hand, do not form a randomly distributed network because of the 1D nature of the MWCNTs. Although it is possible that along with the MWCNT fibre network, the electrical conductivity may be very high, but on the surface of the compression-molded sample, it is unlikely that the measurement is along the connected fibres, especially when the filler content is low. Thus, the measured surface resistivity of the MWCNT-filled sample is higher than those of the GNP-filled samples.

It has been demonstrated in both simulations and experiments that the greater the aspect ratio of the filler material, the smaller the electrical percolation threshold.^45,46^ Due to its great aspect ratio of graphene, the electrical percolation has been shown to be very small, i.e. less than 1 vol.%.^45^ However, this is highly dependent on the matrix material and the method of mixing. In this study, the surface electrical percolation is shown between 1 wt% to 5 wt% for the GNP-filled composite since at 1 wt%, the surface electrical conductivity is still out of the measuring range of the device, while with 5 wt%, the surface electrical conductivity has already been very high. Although it has been reported that the MWCNT-filled composite has even lower percolation threshold,^47^ the results from this study indicate a higher percolation threshold for MWCNT-filled composite than that of the GNP-filled composite. This is mainly caused by the different electrical resistivity measured in this study. The electrical percolation threshold discussed in the literature was the *bulk* electrical resistivity, while the electrical resistivity in this study was measured on the *surface*. The random distribution of the 2D filler in the GNP-filled composites may have resulted in similar electrical properties in both the surface and the bulk of the material, while the 1D MWCNT filler after compression molding may have caused a large difference between the surface and the bulk material. This may also be due to the manufacturing process used, i.e. compression molding, which affected the preferred distribution of the MWCNTs in the matrix and rendered poor filler network formation in the composites.

### Microstructure of the composite coating

**Figure 6.**
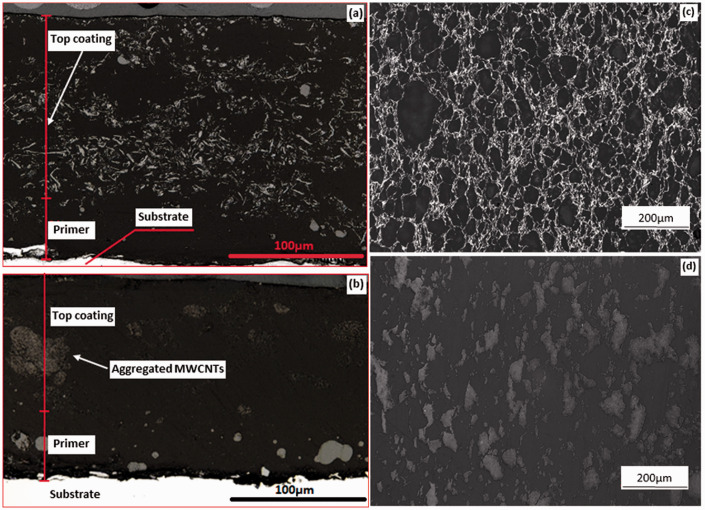
Optical micrographs of the cross-sections of the 10 wt% AO-2 GNP and MWCNT filled composites: (a) AO-2 GNP filled coating, (b) MWCNT filled coating, (c) compression-molded AO-2 GNP filled disk, (d) compression-molded MWCNT filled disk.

Cross-section images of the 10 wt% AO-2 GNP and MWCNT-filled composite coatings are shown in Figure 6(a) and (b), respectively. The GNP-filled coating consists of a ∼150 µm composite top coating, while the MWCNT-filled coating only has about 100 µm top coating. The high aspect ratios of the GNPs and MWCNTs posed challenges to the coating process, i.e., it was more difficult to build up uniform coating layers as compared to graphite-filled PFA coating. For the MWCNT-filled composite, it was only able to achieve ∼100 µm top coating even more coats were applied than the GNP-filled composite. The distributions of the fillers in the composite coatings are significantly different from those after compression molding (Figure 6(c) and 6(d)). The fairly uniform filler network observed in the GNP-filled sample after compression molding (Figure 6(c)) is not seen in the coating (Figure 6(a)). Instead, apparent segregation of the filler particles is observed in the coating. The MWCNT-filled coating (Figure 6(b)) displays extremely uneven distribution of the filler in the matrix, i.e. only a large aggregation of the MWCNTs is observed in the cross-section, which is very different from the compression-molded sample (Figure 6(d)), where, although with agglomerations of the MWCNTs, the distribution is much more uniform.

These discrepancies are caused by the different methods used to prepare the samples. The pressure applied in the compression-molded samples helps the GNPs and MWCNTs reorient in the PFA, which leads to better filler networks. The electrostatic spray coating, on the other hand, is not able to control the orientations of the filler particles during the spray process. The thin and long feature of the MWCNTs makes it very easy to agglomerate when the composite powder was sprayed out from the narrow passage of the spray gun. As a result, the surface finishes of the MWCNT-filled coating are very rough, and the coating thickness is not uniform. All these may deteriorate the corrosion protection ability of the coating.

### Corrosion Resistance

**Figure 7.**
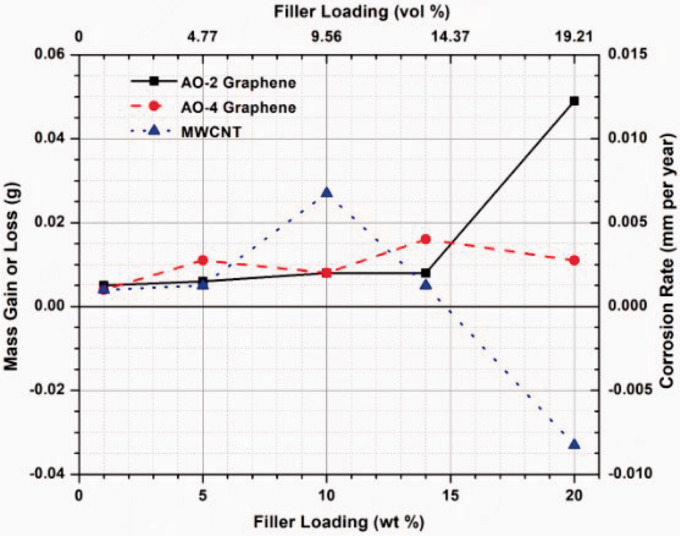
Mass gain or loss (corrosion rate) of the composite coated coupons after 1500 h immersion in 80% sulphuric acid (85°C).

After immersion in an 80% sulfuric acid solution at 85°C for 1500 h, very small mass gain or loss was noticed in the samples, i.e. all was less than 50 mg (0.012 mm/year). It has been shown in previous study^2^ that pure PFA coating without filler showed no corrosion (no mass change) under the same conditions for the same immersion time. Apparently, the filler addition in this study leads to the degradation of the coating quality as all the samples showed mass change after immersion. As shown in Figure 7, when the filler amount reaches 20 wt%, the mass gain or loss is significantly increased for the AO-2 GNP and MWCNT filled coupons, while the mass change for the AO-4 GNP filled coupons is maintained at a very low level (4–15 mg) for all the filler amounts.

**Figure 8.**
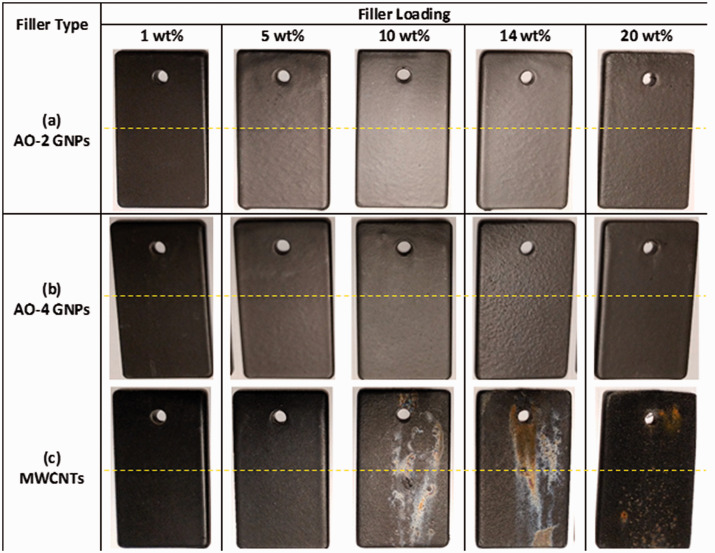
Pictures of the coated coupons after 1500 h immersion in an 80% sulphuric acid solution heated to 85°C. The lines in the figures indicate the positions of immersion: below the line, the sample was immersed; above the line, the sample was not immersed.

Visual examination of the surfaces of the samples revealed that all the GNP-filled samples remained unchanged (Figure 8(a) and (b)), similar to pure PFA samples,^2^ while the MWCNT-filled samples showed apparent discolouration (Figure 8(c)) when the weight fractions reached 10% or more, indicating the onset of corrosion. The long and thin shape of the MWCNT filler in the composite caused difficulties in the electrostatic powder spray process, which resulted in considerably thinner coating thickness as compared to the GNP-filled coatings. On the other hand, the surface finishes of the MWCNT-filled composite coatings are very poor when the filler loading was increased to 10 wt% or more. These make the MWCNT-filled composites coating more susceptible to corrosion than GNP-filled composite coatings. Since corrosion resistance is a critical requirement for condensing heat exchangers, the relatively low corrosion resistance of the MWCNT-filled composites may not fulfill the long-term corrosion resistance requirement for the heat exchangers. For this reason, coating durability testing is not conducted on the MWCNT-filled samples. The GNP-filled composite coatings do not exhibit any sign of discolouration; thus these coatings will be further tested to evaluate the coating adhesion and wear resistance.

### Microscratch Testing

**Figure 9.**
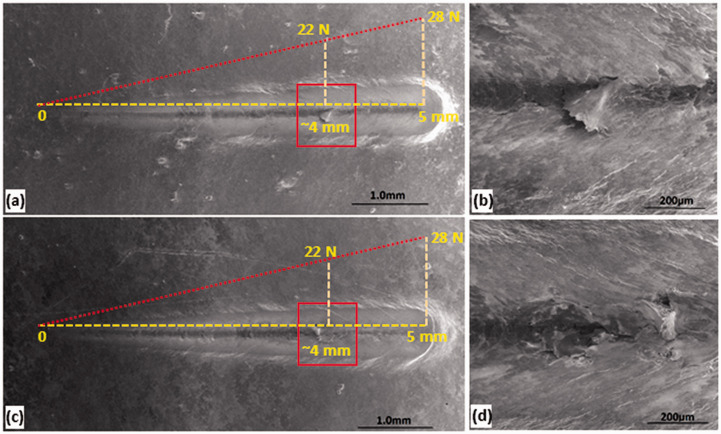
SEM images (secondary electrons) of the scratch tracks: (a) AO-2 GNP filled coating, (b) enlarged view of the boxed area in (a), (c) AO-4 GNP filled coating, (d) enlarged view of the boxed area in (c).

Microscratch tests are performed on samples coated with 20 wt% GNP-filled (both AO-2 and AO-4 grades) composites, and the scratch tracks are shown in Figure 9. Both grades of GNP-filled composites exhibit similar responses to the scratch. As the applied load progressively increases from 0 to 28 N, plastic deformation occurs in the form of grooves and minor material pileups alongside the scratch tracks. At higher applied loads (about 22 N), early signs of failure are observed in both samples (Figure 9(a) and (c)). Higher magnification SEM images (Figure 9(b) and (d)) clearly show the tearing of the coating material, indicating the damage of the composite coating. However, in both samples, the coatings remain adhered to the substrate at all times (no substrate was exposed), indicating excellent bonding to the substrate. This is better than the graphite and boron nitride filled composite coatings from previous research, as higher tendency of coating failure was observed in those coatings^3^. Scratch depth analysis indicates a maximum penetration depth of 100 µm in AO-2 GNP filled sample, and 125 µm in AO-4 GNP filled sample, which are all smaller than the coating thicknesses.

**Figure 10.**
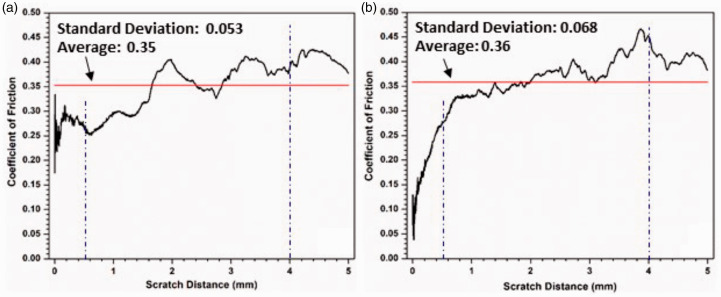
Coefficient of friction (COF) obtained during progressive loading (0 - 28 N) microscratch testing: (a) AO-2 GNP filled coating, (b) AO-4 GNP filled coating.

The coefficient of friction (COF) during microscratch testing is plotted against the sliding distance in Figure 10. The average coefficients of friction of the two samples are quite close, but the COFs show apparent differences between the two samples. The AO-2 GNP filled sample shows a jump of the COF to a high value (∼0.34); it then, fluctuates during the first ∼0.5 mm. Conversely, the COF of the AO-4 GNP filled sample gradually increases from 0.05 to 0.27 during the first ∼0.5 mm. After that, the fluctuation of the COF (around the average) in the AO-2 GNP filled sample is larger than that of the AO-4 GNP filled sample.

Surface topographic measurements on these coatings show that the roughness of the AO-2 GNP filled coating has a considerably larger surface roughness (average: 7.47 ± 0.09 µm, rms: 9.58 ± 0.07 µm) than that of the AO-4 GNP filled coating (average: 5.73 ± 0.26 µm, rms: 7.16 ± 0.39 µm), which leads to more significant COF fluctuations in the AO-2 GNP filled coating. One explanation for the difference in surface roughness is that the AO-2 graphene has much thinner nanoplatelets; thus, the number of nanoplatelets in the matrix is much more than that of the AO-4 graphene under the same weight percentage, which results in higher surface roughness of the coating.

At ∼22 N (4 mm), the AO-2 GNP filled coating experienced partial tear-off damage (Figure 9(b)); whereas the AO-4 GNP filled coating experienced apparent damage in the form of material pileup (Figure 9(d)). The COFs show considerable differences between the two samples: the AO-2 sample displays a lower COF value (∼0.4) than the AO-4 sample (∼0.45), shown in Figure 10.

### Wear testing

**Figure 11.**
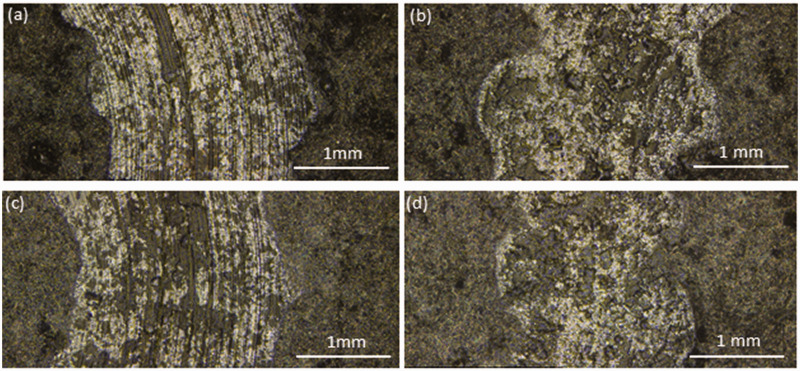
Wear tracks of the 20 wt% GNP-filled composite coatings: (a) AO-2 GNP filler tested at ambient, (b) AO-2 GNP filler tested at 200°C (c) AO-4 GNP filler tested at ambient, (d) AO-4 GNP filler tested at 200°C.

The wear tracks of the 20 wt% GNP-filled composite coatings (both AO-2 and AO-4 grades), tested at both ambient and elevated temperatures, are shown in Figure 11. The samples tested at ambient (Figure 11(a) and (c)) show well-defined tracks (clear arc shape and trace). In contrast, those tested at elevated temperature (Figure 11(b) and (d)) show very diffusive and irregular tracks. This is because at elevated temperatures, the composite coating is softened, and the material is easier to flow than at room temperature. The temperature at the point of contact may even reach the melting point of the polymer,^48,49^ which results in the irregular wear tracks.

**Figure 12.**
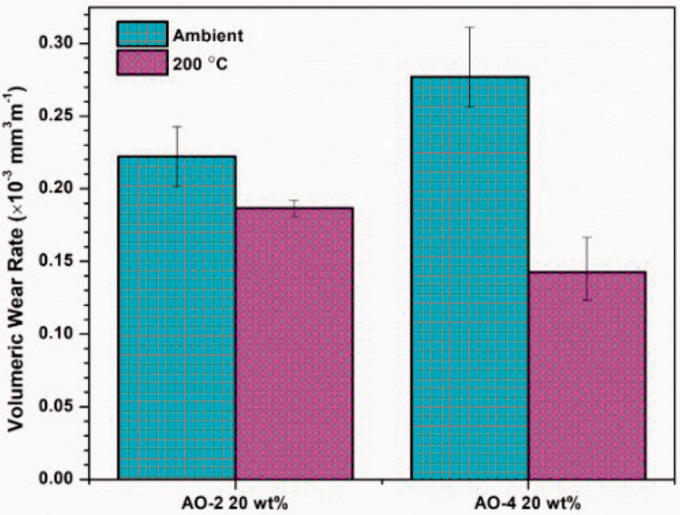
Volumetric wear rates of polymer composite coatings.

Depth analysis of the wear tracks was performed to determine the volumetric loss of material, which, along with the radius of the wear track, allowed for the determination of the volumetric wear rate of the composite coating (Equation (2)). As shown in Figure 12, the room temperature wear rate of the AO-2 GNP-filled composites is considerably lower than that of the AO-4 GNP-filled composite, while at 200°C, the wear rate of the AO-2 GNP-filled composites is higher than that of the AO-4 GNP-filled composite. Since the AO-2 GNPs have much smaller layer thicknesses, the bonding between the filler and matrix is expected to be stronger than that of the thicker AO-4 GNPs. This yields an increase in the hardness of the composite as compared to the AO-4 filled composites, which directly impacts the wear rate.^50,51^

A combination of abrasive and adhesive wear was observed through the microstructural analysis of the wear scars and the counterface balls. A majority of the composite coating was deformed and pushed to the outer edges of the wear scar. This is known as ploughing, and is a mode of abrasive wear that occurs when a hard body (e.g. stainless steel counterface) is worn against a soft material (e.g. polymer composite).^52^ The point of contact between the coating and the counterface may achieve very high temperatures. The polymer matrix is softened at these points and is adhered to the counterface, forming a type of adhesive wear.^52^

**Figure 13.**
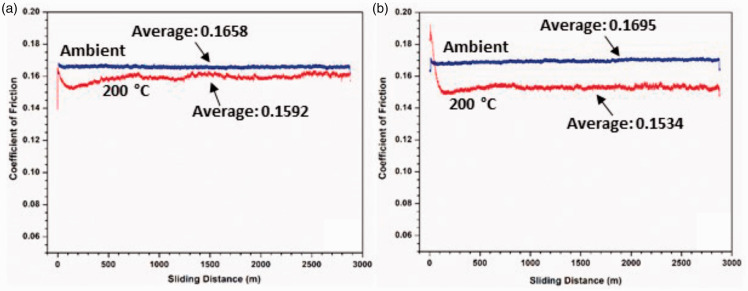
The coefficient of friction versus sliding distance during wear testing at ambient and elevated temperatures: (a) 20 wt% AO-2 GNP-filled coatings, (b) 20 wt% AO-4 GNP-filled coating.

The coefficients of friction of wear testing are shown in Figure 13. It is seen that the coefficients of friction at elevated temperatures are lower than those at room temperature. This is most likely caused by the softening of the polymer surface at higher temperatures, At high temperatures, the polymer is more deformable, resulting in a gliding of the counterface on the polymer, yielding a smooth wear track. COF values for all the tests are between 0.1 and 0.2 throughout the testing, which are within the range of the COFs of PFA on steel.^53^ However, the average COFs of the two composites at room temperature (0.1658 and 0.1695) are about 25% higher than that of graphite-filled composite.^2^ This is likely due to the significantly smaller particle size of the GNPs (5–7 µm flake size) as compared to graphite particles (∼40 µm). Smaller filler particles have demonstrated stronger interfacial bonding with the matrix.^54^ Graphite is known for its lubricating properties; thus, the smaller graphite particles may result in higher COF values as the particles are strongly adhered within the matrix and cannot easily provide a ‘lubricating layer’ on the coating surface during wear. At room temperature, the COF’s of the two GNP-filled samples are essentially the same, whereas at 200°C the COF of the AO-4 filled composite is lower than that of the AO-2 filled sample. Furthermore, the COF values at room temperature are consistently higher than those at 200°C. As mentioned before, at 200°C the composite coating is softened, thus the graphite particles are more easily worn from the coating and may provide a lubricating layer on the coating surface. The slightly larger AO-4 filler particles have a weaker bond within the matrix, and result in a lower COF of the coating during wear. This is consistent with the wear rate as discussed before (Figure 12), i.e. a lower COF will result in a lower wear rate.

From the above analysis, it is seen that the GNP-filled PFA composites show much better thermal properties and corrosion resistance than those filled with MWCNTs; thus, GNPs are better candidates for condensing heat exchanger applications. Of the two graphene grades, although the AO-2 GNP-filled composites show slightly better wear properties at room temperature (but worse at elevated temperature) than the AO-4 GNP-filled composites, other properties, e.g. thermal conductivity, adhesion strength, corrosion resistance, etc., are essentially the same as or slightly worse than the AO-4 GNP-filled composites. Since the AO-4 grade GNPs are much more cost effective, i.e. the price being only a 1/4 of the AO-2 grade, this grade of GNPs is a better choice for the condensing heat exchangers.

## Conclusions

Graphene nanoplatelets (GNPs) and multi-walled carbon nanotubes (MWCNTs) were incorporated into perfluoroalkoxy (PFA) to modify the thermal, electrical, adhesion and wear properties of the composites in order for use in condensing heat exchangers. The characterization of the various properties showed that:

1. Under the same weight percentages, the GNP fillers showed a much larger improvement of the thermal conductivity than the MWCNTs. Of the two grades of GNPs, the AO-4 filled composites exhibited consistently higher thermal conductivities than those filled with the AO-2 grade graphene.
2. The composite-coated coupons only show minimal mass gain or loss (less than 50 mg) after immersion in an 80% sulphuric acid at 85°C for 1500 hours. The MWCNT-filled composite coatings (with ≥ 10 wt% MWCNTs) exhibited apparent discoloration (onset of corrosion), while the GNP-filled coatings did not show such change.
3. None of the GNP-filled composite coatings showed delamination of the coating from the substrate during microscratch testing, although tear-off of the coating material from the scratch track was observed in the AO-2 GNP filled coating.
4. The coefficients of friction of both grades of GNP-filled composite coatings are higher than that of graphite-filled composite, but are within the COF range of PFA on steel. At room temperature, the wear rate of the AO-2 GNP filled coating is lower than that of the AO-4 GNP filled coating, while at 200°C, it is the opposite.
5. AO-4 grade graphene is a better choice for condensing heat exchanger applications, due to its lower cost, higher enhancement of the thermal conductivity, and better resistance to tearing during the microscratch test.
